# To the Editors of the Medical and Physical Journal

**Published:** 1807-11

**Authors:** 


### To the Editors of the Medical and Physical Journal.

GENTLEMEN,

THE Reviewer of "Pethox Parvus," in your last Number, page 384, appears disposed to look upon the inoculation of the small-pox with a more favourable eye than the writer of this note can honestly and conscientiously do.

Some authentic data are yet wanting, to ascertain the precise value of vaccination, compared with variolation. For this purpose, it is necessary to determine the average number of severe and unfortunate cases, under inoculation, not immediately terminating in death; as well as the number of deaths. Is any register of such cases kept at the Small-pox Hospital, or at any other Hospital or Dispensary? If any private practitioner would state the cases of the above kind, which have occurred in his own practice, he would do much service to society.

It is well known, that many of the children in that useful charity, the school for the indigent blind, have lost their sight from the severity of the small-pox:—what proportion do those, rendered blind by the inoculated, bear to those, who have suffered from the casual small-pox?

Can it be ascertained, what is the exact number of deaths, from inoculation of the small-pox, in London? The writer of this has had reason for many years to believe, that among the generality of inoculators, the number of fatal cases has much exceeded one in a hundred.

There is very little doubt, that if the casual small-pox was traced to its source, it would very frequently be found at the Small-pox Hospital, where about two thousand are annually inoculated, and daily carried about the streets while under the eruption. In vol. xiv. p. 311, some very striking cases are published, of the propagation of the casual small-pox, from the careless manner of inoculating, and of suffering the inoculated to intermix with society.

S. M.

*October* 6, 1807.

---

